# Feasibility Study of Using XRV-124 Scintillation Detector for Collinearity Measurement in Uniform Scanning Proton Therapy

**DOI:** 10.14338/IJPT-21-00040.1

**Published:** 2022-04-22

**Authors:** Biniam Tesfamicael, Colton Eckert, Suresh Rana

**Affiliations:** 1 The Oklahoma Proton Center, Oklahoma City, OK, USA; 2 Lynn Cancer Institute, Boca Raton Regional Hospital, Baptist Health South Florida, Boca Raton, FL, USA; 3 Florida International University, Miami, FL, USA

**Keywords:** collinearity, uniform scanning protons, beam coincidence, film, scintillating detector

## Abstract

**Purpose:**

The purpose of this work is to study the feasibility of using an XRV-124 scintillation detector in measuring the collinearity of the x-ray system and uniform scanning proton beam.

**Methods:**

A brass aperture for Snout 10 was manufactured. The center of the aperture had an opening of 1 cm in diameter (4 cm for the film measurements). The 2D kV x-ray images of the XRV-124 were acquired such that the marker inside the detector is aligned to the imaging isocenter. After obtaining the optimal camera settings, a uniform scanning proton beam was delivered for various ranges (12 g/cm^2^ to 28 g/cm^2^ in step size of 2 g/cm^2^). For each range, 10 monitor units (MU) of the first layer were delivered to the XRV-124 detector. Collinearity tests were repeated by using EDR2 and EBT3 films following our current quality assurance protocol in practice. The results from the XRV-124 measurements were compared against the collinearity results from EDR2 and EBT3 films.

**Results and Discussion:**

The collinearity results were evaluated in the horizontal (x) and vertical (y) directions. The average deviation in collinearity in the x-direction was −0.24 ± 0.30 mm, 0.57 ± 0.39 mm, and −0.27 ± 0.14 mm for EDR2, EBT3, and XRV-124, respectively. In the y-direction, the average deviation was 0.39 ± 0.07 mm, 0.29 ± 0.14 mm, and 0.39 ± 0.03 mm for EDR2, EBT3, and XRV-124, respectively.

**Conclusion:**

The measurement results from the XRV-124 and films are in good agreement. Compared to film, the use of the XRV-124 detector for collinearity measurements in uniform scanning protons is more efficient and provides results in real time.

## Introduction

For proton therapy treatment, the alignment of the imaging isocenter to the radiation isocenter is important to ensure that more accurate dose distribution is delivered to the cancer patient [1]. AAPM TG224 report recommends the tolerance of ±1 mm for beam coincidence (also known as collinearity) between x-ray imaging and proton beam [2]. Several research groups have investigated the collinearity in uniform scanning proton therapy [3] and pencil beam scanning (PBS) proton therapy [4–7]. The collinearity can be measured by using film [3] and 2D scintillation detectors such as the Lynx [7].

Recently, the XRV-124 detector (Logos Systems Int'l, Scotts Valley, California)—a 3D scintillation detector—has been more frequently used for the collinearity in PBS proton therapy [1, 4, 5, 8]. Oesten et al [9] evaluated the use of the scintillating detector for image-guided radiation therapy quality assurance (QA) in double scattering and PBS proton beam delivery mode. The XRV-124 can be an efficient device that provides easy setup and high accuracy to perform comprehensive collinearity measurements in PBS proton therapy [1, 4]. Rana et al [1] investigated the accuracy of coincidence between a proton pencil beam and planar kV x-ray imaging system for various energies at different gantry angles. Specifically, their measurement technique included the imaging of the device with kV x-rays and aligning the center of the fiducial with the imaging center [1]. Additionally, Rana et al [1] included a single spot in a given layer such that the intensity of the spots in different layers is varied to avoid the saturation of beam profile. Although the XRV-124 detector has been used for the PBS [1, 4, 5, 8, 9] and double scattering [9] protons, to the best of our knowledge, the feasibility of using the XRV-124 in uniform scanning protons has not been explored.

In uniform scanning proton therapy, a uniform proton dose is delivered to a rectangular scanning area [10]. Since the beam delivery technique in uniform scanning is different from the PBS technique, it is unknown if the XRV-124 can be used for collinearity measurements in uniform scanning proton therapy. Therefore, the primary goal of our study was to investigate the feasibility of using the XRV-124 for collinearity measurements for various energies of uniform scanning proton beams. The XRV-124 results were compared against the film measurements.

## Materials and Methods

The Oklahoma Proton Center is equipped with 4 proton therapy treatment rooms that deliver proton beams, using a uniform scanning beam delivery modality [10]. In this study, to generate a small field proton beam, brass apertures with a small circular opening (of the order of 1 cm in diameter for the XRV-124 measurements and 4-cm diameter for the film measurements) were used. The apertures were designed in such a way that when inserted in the nozzle, the beam's central axis is at the center of the circular opening. The x-ray and proton beam collinearity measurements were performed by using the XRV-124 scintillating detector and film (EDR2 and EBT3).

**Figure 1.**
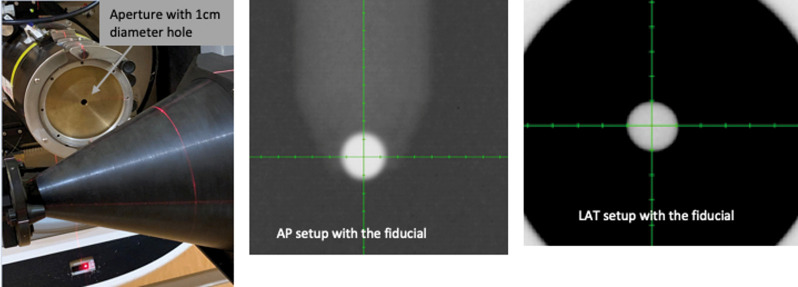
Setup of the XRV-124 for collinearity measurements. The picture on the left is the setup of the detector inside the room with the aperture in the nozzle. The 2 pictures on the right are the 2D image registration with the fiducial. Abbreviations: AP, Anterior-Posterior; LAT, Lateral.

The XRV-124 detector is a cone-shaped scintillating detector with a beam position measurement accuracy of 0.3 mm [10]. When radiation interacts with the scintillator, visible light is emitted at the entrance and exit of the beam. The CCD (charge coupled device) camera captures the visible image from the scintillator, digitizes it, and sends it to the attached computer for analysis [10]. Specifically, the BeamWorks software, version 2.31 (Logos Systems Int'l, Scotts Valley, California) uses this information to calculate the centroids of the 2 profiles obtained from the 2 images and then draws a vector connecting the 2 centroids, that is, the entrance to the exit points. The distance of the vector from the isocenter gives the horizontal and vertical offsets of the beam. For more information on data analysis using BeamWorks software, the readers can refer to the XRV-124 manual [11]. The XRV-124 detector has a rod at its central axis that contains a fiducial at its tip. Using x-ray imaging, the fiducial was first aligned to the center of the imaging system, as shown in **Figure 1**. Once the setup was completed, proton beams of ranges varying from 12 g/cm^2^ to 28 g/cm^2^ were delivered for collinearity measurements. The beam parameters of our current collinearity QA protocol were used in each run. These include a field size of 18 cm × 18 cm and a snout position of 30 cm. For each range, 10 to 12 monitor units (MU) of the first layer were delivered. The deviation between the imaging and beam isocenters was recorded for each run in the BeamWorks software. No user corrections were applied to the XRV-124 data analysis.

**Figure 2.**
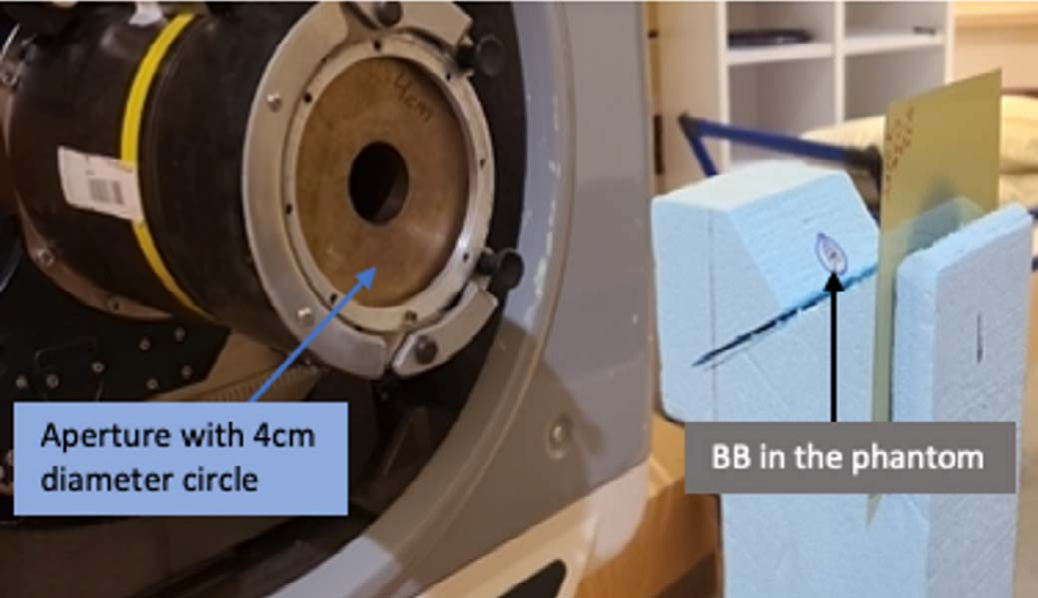
Setup for the film measurement of collinearity. The setup procedure was the same as in the image registration for XRV-124 measurements. Abbreviation: BB, fiducial.

**Figure 3.**
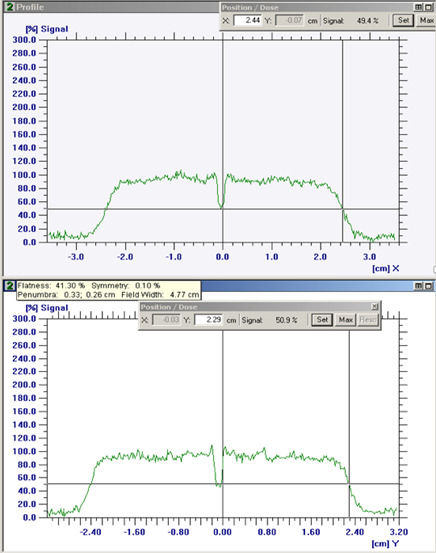
An example of film analysis using the OmniPro I'mRT software. The horizontal and vertical profiles show a dip at the location of the fiducial.

For the same set of proton beam ranges and parameters, collinearity measurements were also performed by using both EDR2 and EBT3 films. These measurements were carried out as based on our current institutional protocol. A phantom with a fiducial is first set up by using x-ray imaging, as shown in **Figure 2**. At the completion of the setup, the fiducial is aligned to the center of the imaging system. The phantom has space for a film behind the fiducial, downstream the beam, at a distance of about 3.5 cm. Proton beams with the above-mentioned parameters were run, and only 10 MU of the first layer was delivered for each range. The films were then scanned and analyzed with the OmniPro I'mRT software (IBA Dosimetry, Schwarzenbruck, Germany) to calculate the deviation between the x-ray and proton beam isocenters. **Figure 3** shows an example of profile analysis from the OmniPro I'mRT software. Specifically, the horizontal and vertical profiles show a dip at the location of the fiducial. As part of the analysis, the coordinate of the center of the profile is calculated. The location of the center of the dip relative to the center of the profile gives the offset of the beam isocenter from the x-ray imaging system. The results of the XRV-124 and film measurements are discussed in the “Results and Discussion” section below.

## Results and Discussion

**Figure 4.**
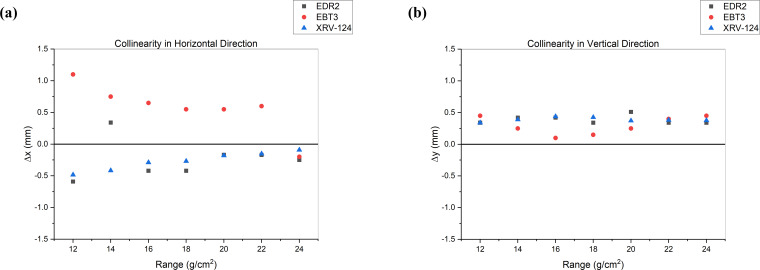
Plot of deviation (Δx, Δy) of imaging and beam collinearity for different beam ranges in both horizontal and vertical directions.

The use of XRV-124 for collinearity measurements has been well studied and implemented by many researchers, mainly for PBS [1, 4]. In this study, we investigated the application of the XRV-124 scintillating detector for x-ray and proton beam coincidence measurement in uniform scanning proton beam delivery technique. The film has been a detector of choice for collinearity measurements in many cancer centers. In our investigation, we used film as a reference to verify the accuracy of the measurement results from the XRV-124. The deviation between the imaging and beam collinearity measured by the different detectors is shown in **Figure 4**.

**Figure 4** shows an excellent agreement between the XRV-124 and film measurements. In the vertical direction, the deviations between the XRV and film measurement results are very minimal. In the horizontal direction, there is very good agreement between the XRV-124 and the EDR2 when compared against the agreement between XRV-124 and EBT3. The minor deviation between the results of XRV-124 and film can be attributed to different factors such as film processing and method of analysis. The results of our study imply that the XRV-124 can be used for imaging and beam collinearity measurements, replacing film with acceptable accuracy. Implementation of the XRV-124 for collinearity measurement can reduce the cost of film, film processing equipment like scanners, and analysis software from a third-party vendor. Moreover, the XRV-124 provides the results in real time, thus providing immediate feedback on the measurement to make a quick clinical decision. Film measurements, however, require a prolonged processing step. In the long run, the use of film does not appear to be cost-effective to a center with multiple rooms performing the collinearity measurements in multiple gantry angles on a monthly and annual basis. Hence, the use of the XRV-124 for collinearity measurements can be proven to be more efficient, accurate, and cost-effective in the long run.

**Figure 5.**
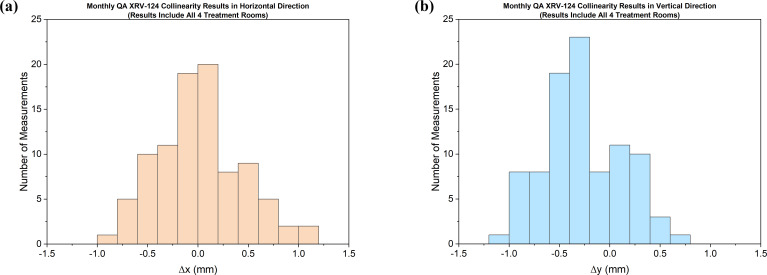
Monthly QA collinearity measurement results for all 4 treatment rooms using the XRV-124 detector. The tolerance limit is within ±1 mm. Abbreviation: QA, quality assurance.

After the thorough validation measurements, we have been using the XRV-124 detector for collinearity measurements in uniform scanning proton beams in all our treatment rooms. The ease of the setup and the quick acquisition of the data makes the XRV detector a much-preferred device for our monthly as well as annual QA. The data displayed in **Figure 5** are the monthly QA measurement results from all the treatment rooms. Almost all the XRV-124 measurement results are within the tolerance limit (±1 mm) of our QA protocol. The collinearity measurements in our study included gantry angles of 30° and 90° only. This is a limitation of our study. With the availability of XRV-124 at our center, our goal is to test the collinearity measurements at various angles in our gantry room.

## Conclusion

The XRV-124 scintillating detector can be used for imaging and beam isocenter collinearity measurement in a uniform scanning proton beam delivery technique. The measurement results from the scintillating detector were validated against film measurements. The results obtained using the XRV-124 and films were generally in good agreement.
